# P64 Improving antimicrobial stewardship in critical care through a structured 72 h review: a quality improvement study

**DOI:** 10.1093/jacamr/dlag102.070

**Published:** 2026-06-26

**Authors:** Alexandra Muir, Claire McCue, Scott Gillen

**Affiliations:** Critical Care, Glasgow Royal Infirmary, Greater Glasgow and Clyde, Glasgow, UK; Critical Care, Glasgow Royal Infirmary, Greater Glasgow and Clyde, Glasgow, UK; Critical Care, Glasgow Royal Infirmary, Greater Glasgow and Clyde, Glasgow, UK

## Abstract

**Background:**

AMS is a key priority in critical care, where antimicrobial use is high. The Extended Prevalence of Infection in Intensive Care Study (2007), a global ICU point prevalence study, found 71% of patients were prescribed antibiotics, and treatment courses are frequently prolonged, contributing to antimicrobial resistance. The *Start Smart Then Focus* strategy, developed by NHS England and endorsed by the Faculty of Intensive Care Medicine, promotes early review of antimicrobial therapy however this is inconsistently achieved. Baseline data from our critical care unit suggested variable documentation of indications and durations for antibiotic prescriptions. This project formed part of a wider unit and health board level initiative to improve AMS and aimed to optimize antimicrobial use through improved documentation and prescribing practices.

**Objectives:**

To improve antimicrobial prescribing in critical care by implementing a structured 72-h review (‘pause’), with a focus on increasing documentation of indication and duration and promoting timely reassessment of therapy in line with antimicrobial stewardship (AMS) principles.

**Methods:**

Baseline antimicrobial prescribing practices were audited using point prevalence surveys conducted in June, August, and October 2025. Data collected included the proportion of patients prescribed antimicrobials, documentation of indication at 72 h, and documentation of intended duration. A quality improvement intervention was developed introducing a structured 72 h antimicrobial review, the ‘72 h pause’. This was embedded into the electronic microbiology round documentation. It included mandatory prompts requiring clinicians to document duration and to select a 72 h review outcome (continue, amend, or cease). The intervention was supported by departmental teaching sessions and posters to reinforce AMS principles and promote engagement. A repeat point prevalence survey was conducted in March 2026 following implementation.

**Results:**

At baseline, between 58.8% and 75% of patients were prescribed at least one antimicrobial. Documentation of key stewardship elements was inconsistent. Intended duration was documented in the microbiology notes in 46.7% of cases in June, 55.5% in August, and 45.4% in October. Following implementation of our intervention, this improved to 84.6%. This was also translated into an improvement in defined duration on the actual prescriptions, with pre-intervention audits showing 17.6%, 18.2%, and 23.5%, respectively, having it documented. Following the intervention, we saw an improvement, with 55% of patients having a duration stated on the prescription. No improvement was seen with documentation of indication. Pre-intervention data showed 41.2%, 9.1%, and 0%; post-intervention this remained low at 10%.

**Conclusions:**

The introduction of a structured 72 h pause, supported by changes to electronic documentation and targeted education, significantly improved documentation of duration and facilitated earlier review of antimicrobial therapy in critical care. There was no improvement in documentation of indication, which may be attributed to the absence of a clear prompt or dedicated location, relying instead on clinicians using free-text field on prescription. These findings suggest that integrating AMS principles into electronic note formats and prescribing systems can drive improvements in prescribing behaviour. Increased education and introduction of a dedicated field for indication may further improve compliance. Ongoing point prevalence audits are required to assess sustained improvement in documentation and prescribing practices.

